# Human platelet antigen-3 polymorphism as a risk factor for rheumatological manifestations in hepatitis C

**DOI:** 10.1590/0037-8682-0210-2019

**Published:** 2020-01-27

**Authors:** Natália Bronzatto Medolago, Adriana Camargo Ferrasi, Oswaldo Melo da Rocha, Maria Inês de Moura Campos Pardini, Rejane Maria Tommasini Grotto, Aline Faria Galvani, Giovanni Faria Silva

**Affiliations:** 1 Universidade Estadual Paulista, Departamento de Clínica Médica, Faculdade de Medicina de Botucatu, Botucatu, SP, Brasil.; 2 Universidade Estadual Paulista, Departamento de Bioprocessos e Biotecnologia, Faculdade de Ciências Agronômicas, Botucatu, SP, Brasil.

**Keywords:** Hepatitis C, Human platelet antigens, Polymorphism, Rheumatological manifestations

## Abstract

**INTRODUCTION:**

Hepatitis C virus (HCV) infection is involved in the pathogenesis of autoimmune and rheumatic disorders. Although the human platelet antigens (HPA) polymorphism are associated with HCV persistence, they have not been investigated in rheumatological manifestations (RM). This study focused on verifying associations between allele and genotype HPA and RM in patients with chronic hepatitis C.

**METHODS:**

Patients (159) with chronic hepatitis C of both genders were analyzed.

**RESULTS:**

Women showed association between HPA-3 polymorphisms and RM.

**CONCLUSIONS:**

An unprecedented strong association between rheumatological manifestations and HPA-3 polymorphism, possibly predisposing women to complications during the disease course, was observed.

Chronic hepatitis C virus (HCV) infection is a worldwide public health problem with a global prevalence of 2-3%^1^. In addition to being a frequent cause of chronic liver diseases such as hepatitis, cirrhosis, and hepatocellular carcinoma, it is also involved in the pathogenesis of various autoimmune and rheumatic disorders such as arthritis, vasculitis, sicca syndrome, porphyria cutanea tarda, lichen planus, nephropathies, thyroid diseases, lung fibrosis, among others^1^ ^,^ ^2^ ^,^ ^3^. Even though the rheumatic disorders are common among the extrahepatic manifestations, the mechanisms involved in the onset of these symptoms as well as the associated genetic factors are yet to be understood completely.

Host genetic factors such as the the human platelet antigens (HPA) polymorphism are also known to be associated with the infection and persistence of HCV^4^ ^,^ ^5^. However, there is no evidence of its relation to rheumatological manifestations.

HPAs result from the polymorphisms in the genes encoding surface glycoproteins of platelets, endothelial cells, and fibroblasts^6^ ^,^ ^7^, and are commonly involved in rheumatological diseases. Considering that the fibroblasts express HPA, these proteins could be involved in rheumatological manifestations. Thus, the aim of this study was to verify associations between allele and genotype HPA-1, -3, and -5 polymorphisms and rheumatological manifestations in patients with chronic hepatitis C.

A total of 159 individuals aged between 18 and 80 years, of both genders and affected by chronic hepatitis C, assisted at the Viral Hepatitis Outpatient Clinic of Botucatu Medical School, Unesp, Brazil, were included in this study. We only considered detectable HCV-RNA cases, with identification of HCV genotype, no previous hepatitis C treatment (*naïve* patients), with known fibrosis stage or clinical diagnosis of cirrhosis by image. Patients with HBV/HIV co-infection, chronic renal insufficiency, liver or renal transplantation, liver diseases, and other diffuse connective tissue diseases, including rheumatoid arthritis, according to Rheumatoid Arthritis Classification Criteria (ACR-EULAR 2010), were excluded^8^.

Clinical symptoms, such as presence of paresthesia sensations, Raynaud's phenomenon, cutaneous alterations, subcutaneous nodule, myalgia, muscle weakness, non-mechanical low back pain, arthralgia, arthritis, and other rheumatological manifestations were considered as rheumatological manifestations in this study. Laboratorial parameters evaluated were rheumatoid factor (qualitative and semi-quantitative) and anti-CCP (semi-quantitative) using Reumalatex kit (Labtest Diagnostica S/A, Lagoa Santa, MG, Brazil) and QUANTA LiteTM CCP3.1 kit (INOVA Diagnostic Inc., San Diego, CA, USA), respectively, according to the manufacturer’s instructions.

Deoxyribonucleic acid (DNA) was isolated from the total blood using the Wizard^®^ Minipreps DNA Purification System and used to genotype HPA-1 and HPA-3 with polymerase chain reaction-sequence-specific primers (PCR-SSP), as described by Klüter et al^9^. HPA-5 was genotyped using polymerase chain reaction-restriction fragment length polymorphism (PCR-RFLP), as described by Kalb et al^10^.

The association analysis between the categorical variables was performed using the χ^2^ or Fisher’s exact test. Student's t-test was used for comparing the mean ages. Logistic regression was used to categorize the risk of the association among the groups. Odds ratio values with 95% conﬁdence interval were also calculated. *P*≤ 0.05 was considered statistically significant.

This study was approved by the Ethics Committee on Research of Sao Paulo State University (Protocol 3727/2010), and conforms to the provisions of Helsinki Declaration of 1964, as revised in 1975, 1983, 1989, 1996, and 2000. All the participants of this study signed the individually informed consent forms. 

Out of 159 individuals, 87 (54.7%) were men and 72 (45.3%) were women. The median and mean ages were 49 years (24 to 76 years) and 48.7 years, respectively.

Rheumatological manifestations were present in 72.3% of the patients. Table 1 shows the demographic and clinical characteristics related to rheumatic involvement. An association between female gender and development of rheumatological manifestations (*P* = 0.0201) was observed. This association was maintained when the data was subjected to multivariate logistic regression analysis (*P* = 0.0381). It is well-known that the rheumatic diseases are more prevalent in women, regardless of other concomitant clinical conditions^11^. The correlation between rheumatic manifestation and female gender was already observed in a research conducted with Egyptian population affected by chronic hepatitis C^12^. In addition, Cacoub et al^13^ showed that more than 70% of the HCV-infected patients showed extrahepatic manifestations involving primarily joints, muscles, and skin, which as per our findings, were also associated to female gender.

**TABLE 1:** Clinical and demographic characteristics of the population with chronic hepatitis C, distributed by presence or absence of rheumatological manifestations.

Variables	Presence of rheumatological	Absence of rheumatological	*P-value*
	manifestations	manifestations	
	n = 115 (%)	n = 44 (%)	
Age (years), mean	49.6 ± 10.0	46.3 ± 10.3	0.0672^†^
**Sex**			
Male	56 (64.4)	31 (35.6)	0.0201
Female	59 (81.9)	13 (18.1)	
**Ethnicity**			
White	102 (72.3)	39 (27.7)	1.0000
Non-white	13 (72.2)	5 (27.8)	
HCV Genotype			
1	80 (73.4)	29 (26.6)	0.7042
Not 1	35 (70.0)	15 (30.0)	
Fibrosis^‡^			
Absent (F0)	3 (75.0)	1 (25.0)	0.0519
Moderate (F1, F2)	48 (64.0)	27 (36.0)	
Advanced (F3)	20 (69.0	9 (31.0)	
Cirrhosis	44 (86.3)	7 (13.7)	

Fisher's exact test or Chi-square test (**χ** ^2^); *P* ≤ 0.05 is considered a statistically significant relation; ^†^T-test; ^‡^Histological grouping.

Genotype and allele frequencies of HPA-1, -3, and -5 were distributed according to the presence or absence of rheumatological manifestations. There was no significant association observed among the patients. However, upon considering the gender (Table 2 and Table 3), the females showed a significant association between rheumatological manifestation and allele HPA-3a (OR = 3.83, 95% CI = 1.60-9.22, and *P* = 0.0044) and HPA-3a3a (OR = 6.98, 95% CI = 1.42-34.31, and *P* = 0.0125). Moreover, a risk was also observed for HPA-1a1b (OR = 7.67, 95% CI = 0.93-63.02, and *P* = 0.0482). On the contrary, HPA-3b3b was protective (OR = 0.21, 95% CI = 0.47-0.93, and *P* = 0.0496) for rheumatological manifestations.

**TABLE 2:** Genotype and allele frequencies of HPA-1, -3, and -5 in women with chronic hepatitis C, distributed by the presence and absence of rheumatological manifestations.

HPA	Presence of	Absence of	Statistical Analysis	
	rheumatological	rheumatological		
	manifestations	manifestations		
**Alleles**	**2n = 118 (%)**	**2n = 26 (%)**	**OR (CI 95%)**	***P-value***
1a	93 (80.2)	23 (19.8)	0.49 (0.13 - 1.75)	0.4111
1b	25 (89.3)	3 (10.7)	2.06 (0.57 - 7.43)	
3a	87 (88.8)	11 (11.2)	3.83 (1.60 - 9.22)	0.0044
3b	31 (67.4)	15 (32.6)	0.26 (0.11 - 0.63)	
5a	99 (79.8)	25 ( 20.2)	0.21 (0.03 - 1.63)	0.1257
5b	19 (95.0)	1 (5.0)	4.80 (0.61 - 37.60)	
**Genotypes**	**n = 58 (%)**	**n = 13 (%)**	**OR (CI 95%)**	***P-value***
1a/1a	35 (76.0)	11 (24.0)	0.27 (0.05 - 1.31)	0.1155
1a/1b	23 (95.8)	1 (4.2)	7.67 (0.93 - 63.02)	0.0482
1b/1b	1 (50.0)	1 (50.0)	0.21 (0.01 - 3.55)	0.3306
3a/3a	33 (94.3)	2 (5.7)	6.98 (1.42 - 34.31)	0.0125
3a/3b	21 (75.0)	7 (25.0)	0.47 (0.14 - 1.60)	0.3460
3b/3b	5 (55.6)	4 (44.4)	0.21 (0.47 - 0.93)	0.0496
5a/5a	42 (77.8)	12 (22.2)	0.21 (0.03 - 1.71)	0.1628
5a/5b	15 (93.8)	1 (6.2)	4.09 (0.49 - 34.20)	0.2723
5b/5b	2 (100.0)	0 (0)	1.17 (0.05 - 25.91)	1.0000

Fisher's exact test; **OR:** odds ratio; **CI:** confidence interval; *P* ≤ 0.05 is considered a statistically significant relation.

**TABLE 3:** Genotype and allele frequencies of HPA-1, -3, and -5 in men with chronic hepatitis C, distributed by the presence and absence of rheumatological manifestations.

HPA	Presence of	Absence of	Statistical Analysis	
	rheumatological	rheumatological		
	manifestations	manifestations		
**Alleles**	**2n = 112 (%)**	**2n = 62 (%)**	**OR (CI 95%)**	***P-value***
1a	97 (66.4)	49 (33.6)	1.72 (0.76 - 3.90)	0.2030
1b	15 (53.6)	13 (46.4)	0.58 (0.26 - 1.32)	
3a	76 (62.8)	45 (37.2)	0.80 (0.40 - 1.58)	0.6067
3b	36 (67.9)	17 (32.1)	1.25 (0.63 - 2.50)	
5a	99 (64.7)	54 (35.3)	1.13 (0.44 - 2.89)	0.8115
5b	13 (61.9)	8 (38.1)	0.89 (0.35 - 2.27)	
**Genotypes**	**n = 56 (%)**	**n = 31 (%)**	**OR (CI 95%)**	***P-value***
1a/1a	44 (69.8)	19 (30.2)	2.32 (0.88 - 6.08)	0.1318
1a/1b	9 (45.0)	11 (55.0)	0.35 (0.13 - 0.97)	0.0612
1b/1b	3 (75.0)	1 (25.0)	1.70 (0.17 - 17.07)	1.0000
3a/3a	26 (63.4)	15 (36.6)	0.92 (0.38 - 2.23)	1.0000
3a/3b	24 (61.5)	15 (38.5)	0.80 (0.33 - 1.93)	0.6577
3b/3b	6 (85.7)	1 (14.3)	3.60 (0.41 - 31.39)	0.4135
5a/5a	44 (65.7)	23 (34.3)	1.28 (0.46 - 3.56)	0.7908
5a/5b	11 (57.9)	8 (42.1)	0.70 (0.25 - 1.99)	0.5907
5b/5b	1 (100.0)	0 (0)	1.70 (0.07 - 43.09)	1.0000

Fisher's exact test; **OR:** odds ratio; **CI:** confidence interval; *P* ≤ 0.05 is considered a statistically significant relation.

In this context, it is noteworthy that HPA-1 and HPA-3 are located in the same glycoprotein complex (GPIIb-IIIa) expressed in both endothelial cells and fibroblasts^7^, which are the cells commonly involved in rheumatological diseases. However, additional studies involving other populations are necessary to confirm these data and to improve the understanding of the mechanisms involved in rheumatic manifestations in chronic HCV infection. Similar to the well-established association of human leukocyte antigens (HLA) and diseases, studies involving HPA may also contribute towards the identification of clinically important molecular markers, thereby aiding in understanding the pathophysiological mechanisms involved in the diseases.

Our study is the first report of a strong association between rheumatological manifestations and HPA-3 polymorphism, which may explain the possible predisposition of women to complications during the course of chronic hepatitis C.
